# DFAST: a flexible prokaryotic genome annotation pipeline for faster genome publication

**DOI:** 10.1093/bioinformatics/btx713

**Published:** 2017-11-02

**Authors:** Yasuhiro Tanizawa, Takatomo Fujisawa, Yasukazu Nakamura

**Affiliations:** Center for Information Biology, National Institute of Genetics, Research Organization of Information and Systems, 1111 Yata, Mishima, Japan

## Abstract

**Summary:**

We developed a prokaryotic genome annotation pipeline, DFAST, that also supports genome submission to public sequence databases. DFAST was originally started as an on-line annotation server, and to date, over 7000 jobs have been processed since its first launch in 2016. Here, we present a newly implemented background annotation engine for DFAST, which is also available as a standalone command-line program. The new engine can annotate a typical-sized bacterial genome within 10 min, with rich information such as pseudogenes, translation exceptions and orthologous gene assignment between given reference genomes. In addition, the modular framework of DFAST allows users to customize the annotation workflow easily and will also facilitate extensions for new functions and incorporation of new tools in the future.

**Availability and implementation:**

The software is implemented in Python 3 and runs in both Python 2.7 and 3.4—on Macintosh and Linux systems. It is freely available at https://github.com/nigyta/dfast_core/under the GPLv3 license with external binaries bundled in the software distribution. An on-line version is also available at https://dfast.nig.ac.jp/.

**Supplementary information:**

Supplementary data are available at *Bioinformatics* online.

## 1 Introduction

Most scientific journals require newly obtained sequence data to be deposited in the International Nucleotide Sequence Database Collaboration (INSDC) as a condition of publication (Cochrane *et al.*, 2016). However, submission of annotated genomes to public databases remains a burden for researchers. The NCBI provides an annotation service called Prokaryotic Genome Annotation Pipeline (PGAP) (Tatusova *et al.*, 2016) incorporated in its submission system, but it is only available for GenBank submitters. The on-line server Microbial Genome Annotation Pipeline (MiGAP) (Sugawara *et al.*, 2009) partly supports DDBJ submission; however, it requires extensive manual revision. To address these issues, we recently developed a web-based pipeline called DDBJ Fast Annotation and Submission Tool (DFAST), aiming to assist users to submit their genomes to DDBJ (Tanizawa *et al.*, 2016). The original version of DFAST employs the lightweight command-line program Prokka (Seemann, 2014) as an annotation engine, combined with curated reference databases and a graphical user interface to create submission files to DDBJ.

Here, we report a new implementation of the background engine of DFAST, which is called DFAST-core to differentiate it from its web version. The new version features unique functions, such as pseudogene annotation and orthologous assignments between reference genomes. DFAST-core is also available as a standalone program, providing a flexible local annotation platform. Hereinafter, we simply refer to it as DFAST in this report.

## 2 Materials and methods

**Fig. 1:**
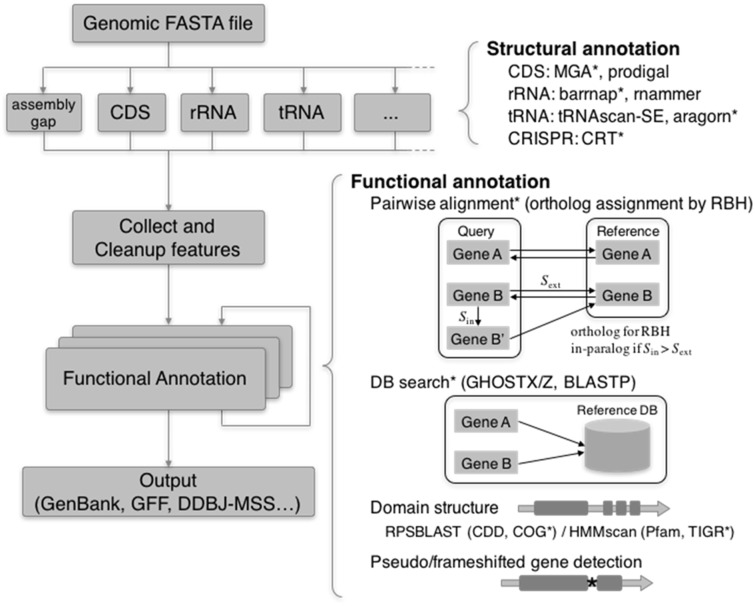
DFAST annotation workflow. Items marked with asterisks are included in the default workflow

DFAST accepts a FASTA-formatted file as a minimum required input, and users can customize parameters, tools and reference databases by providing command line options or defining an original configuration file (see Supplementary Notes for more details). The workflow is mainly composed of two annotation phases, i.e. structural annotation for predicting biological features such as CDSs, RNAs and CRISPRs, and functional annotation for inferring protein functions of predicted CDSs. Figure 1 shows a schematic depiction of the pipeline. Each annotation process is implemented as a module with common interfaces, allowing both flexible annotation workflows and extensions for new functions in the future.

In the default configuration, functional annotation will be processed in the following order:

1. Orthologous assignment (optional) All-against-all pairwise protein alignments are conducted between a query and each reference genome. Orthologous genes are identified based on a Reciprocal-Best-Hit approach. It also conducts self-to-self alignments within a query genome, in which genes scoring higher than their corresponding orthologs are considered in-paralogs and assigned with the same protein function. This process is effective in transferring annotations from closely related organisms and in reducing running time.
2. Homology search against the default reference database DFAST uses GHOSTX as a default aligner, which runs tens to hundred times faster than BLASTP with similar levels of sensitivity where E-values are less than 10^−6^ (Suzuki *et al.*, 2014). Users can also choose BLASTP. For accurate annotation, we constructed a reference database from 124 well-curated prokaryotic genomes from public databases. See Supplementary Data for the breakdown of the database.
3. Pseudogene detection CDSs and their flanking regions are re-aligned to their subject protein sequences using LAST, which allows frameshift alignment (Kiełbasa *et al.*, 2011). When stop codons or frameshifts are found in the flanking regions, the query is marked as a possible pseudogene. This also detects translation exceptions such as selenocysteine and pyrrolysine.
4. Profile HMM database search against TIGRFAM (Haft *et al.*, 2013) It uses hmmscan of the HMMer software package.
5. Assignment of COG functional categories RPS-BLAST and the rpsbproc utility are used to search against the Clusters of Orthologous Groups (COG) database provided by the NCBI Conserved Domain Database (Marchler-Bauer *et al.*, 2017).

DFAST output files include INSDC submission files as well as standard GFF3, GenBank and FASTA files. For GenBank submission, two input files for the tbl2asn program are generated, a feature table (.tbl) and a sequence file (.fsa). For DDBJ submission, DFAST generates submission files required for DDBJ Mass Submission System (MSS) (Mashima *et al.*, 2017). In particular, if additional metadata such as contact and reference information are supplied, it can generate fully qualified files that are ready for submission to MSS.

While the workflow described above is fully customizable in the stand-alone version, only limited features are currently available in the web version, e.g. orthologous assignment is not available. As a merit of the web version, users can curate the assigned protein names by using an on-line annotation editor with an easy access to the NCBI BLAST web service. We also offer optional databases for specific organism groups (*Escherichia coli*, lactic acid bacteria, bifidobacteria and cyanobacteria). They are downloadable from our web site and can be used in the stand-alone version. We are updating reference databases to cover more diverse organisms.

## 3 Results and discussion

We annotated the genome of *Escherichia coli* O26: H11 str. 11368 using DFAST, Prokka and MiGAP, and compared the results to the INSDC data manually curated by original submitters (deposited in the NCBI Assembly Database under GCA_000091005.1) and the RefSeq data annotated using PGAP (GCF_000091005.1), as summarized in Table 1.

**Table 1.** Comparison of annotation results of *E.coli* O26: H11 str. 11368

Data source/Annotation tool	INSDC^a^	RefSeq^b^	DFAST	Prokka	MiGAP
Total CDS	5795	6243	5740	5759	5721
*Pseudogene*^c^	276	337 (250/87)	344 (158/186)	[30^d^]	—
*Selenoprotein*	3	1	3	—	—
*With COG number*	—	—	3965	—	4392
*Unknown function*	1203	1514	1347	2068	418
tRNA	101	101	105	105	100
rRNA	22	22	22	22	22
CRISPR array	—	2	2	2	—
Running time	—	—	3 m 27 s	3 m 20 s	4 h 43 m

*Note*: Numbers represent annotated features and running time. DFAST and Prokka were run on a 4-core Macintosh laptop with default settings.

a Original annotation by submitters (GCA_000091005.1).

b Annotated by PGAP (GCF_000091005.1).

c Numbers in parentheses denote internal stop codon/frameshift and partial genes, respectively.

d Candidates for pseudogenes are mentioned in the log file, not in the result.

Our simple strategy to find pseudogenes depends on the accuracy of reference databases. However, when references from close relatives are available, DFAST outperforms other tools. Among 158 CDSs in which internal stop codons or frameshifts were identified, 123 were found to be consistent with the INSDC data (78%). Although the comparison is not straightforward as annotation formats are different, 97 out of 250 identified by PGAP were consistent (39%). Notably, DFAST succeeded in annotating all 3 selenoproteins present in the query genome.

Another major advantage of our pipeline is its speed. The running time of DFAST is comparable with that of Prokka, yet the default reference database of DFAST (417 922 sequences in total) is 20 times larger than that of Prokka (18 276 sequences). This is mostly attributable to the efficient algorithm of GHOSTX. If BLASTP is used instead, running time will increase up to 40 min under the same condition. In accordance with the database size, the number of genes with assigned function was larger than Prokka, although smaller than MiGAP, which conducts sequence search against a more comprehensive database such as UniProtKB/TrEMBL.

In general, DFAST performs well with the default settings on well-characterized organisms, such as *Actinobacteria*, *Firmicutes* and *Proteobacteria*. The annotation of the genomes from less-studied species, for which references of close relatives are not present in the default database, may contain relatively large number of uncharacterized genes. In such cases, providing additional references will improve the results as demonstrated in Supplementary Notes.

## Supplementary Material

Supplementary Data
