# Supplementary material for: DFAST: a flexible prokaryotic genome annotation pipeline for faster genome publication
Source: Bioinformatics. 2017 Nov 2;34(6):1037–9. doi: 10.1093/bioinformatics/btx713 (PMC5860143; doi:10.1093/bioinformatics/btx713)
Supplement: Supplementary Data [file btx713_supp.zip › btx713-suppl_data/SupplementaryNotes.docx]

**DFAST: a flexible prokaryotic genome annotation pipeline for faster genome publication**

**Supplementary Notes**

# Reference database

The default reference database mainly consists of protein sequences from 124 representative prokaryotic genomes. Among them, 115 were bacterial genomes labeled as ‘reference genomes’ in the RefSeq database (https://www.ncbi.nlm.nih.gov/refseq/about/prokaryotes/#referencegenome). We also selected 9 archaeal genomes from RefSeq with priority given to well-characterized complete genomes. For consistent protein naming, we preferentially used recommended names by UniProtKB or protein names used in the RefSeq non-redundant proteins (https://www.ncbi.nlm.nih.gov/refseq/about/nonredundantproteins/), if available, rather than the original submitters’ name. Also, we added selenocysteine/pyrrolysine-containing proteins from SwissProt. Protein names were partly curated automatically; locus tags were removed if included in the names, proteins with unknown function were renamed as ‘hypothetical protein’, and ‘partial’ or ‘fragmented’ sequences were excluded.

The current version of the reference database (ver. 1.1) includes 417,922 protein sequences with total residues of 131,673,294. The breakdown of the reference sequences is shown in Table S1.

# Examples of annotation results

In addition to the *Escherichia coli* genome annotation described in the main text, we tested DFAST on the following organisms: *Lactobacillus* *parakefiri* as an example for draft genome annotation and for comparison with the former version of DFAST, *Candidatus* Brocadia sinica as an example for less well-characterized organisms, *Methanosarcina barkeri* as an example for archaeal genome annotation. We compared the results with annotations from different platforms including PGAP, Prokka, and MiGAP. Unless noted otherwise, DFAST and Prokka were executed on a Macintosh notebook with 4 CPU cores. INSDC and RefSeq data were obtained from the NCBI assembly database. The results are summarized in Table S2–S3.

## *Lactobacillus* *parakefiri*

In our recent study, we annotated the draft genome of *L. parakefiri* JCM 8573^T^ (2.49 Mb, GCA_002157585.1) using the former version of DFAST with a custom reference database for lactic acid bacteria (Tanizawa *et al.*, 2017). *L. parakefiri* is a heterofermentative member of the genus *Lactobacillus*, which is the most diverse group of lactic acid bacteria consisting of nearly 200 known species. For finer-grained annotation, we collected protein sequences mainly from complete genomes of *Lactobacillus* and constructed a manually-curated reference database following the NCBI protein naming guideline (<https://www.ncbi.nlm.nih.gov/genbank/genomesubmit_annotation/#CDS)> (Tanizawa *et al.*, 2016). As a result, we obtained a submission file that was acceptable to DDBJ almost without further refinement (Table S2-A). Here, we present the re-annotation of this genome using the new version of DFAST (C, D).

Since *L. parakefiri* has a small and simple genome, the numbers of predicted genes were similar regardless of annotation methods. The large number of rRNAs identified in the RefSeq records was due to the ones predicted at the edges of the contigs. DDBJ does not recommend such partial features to be submitted unless there is some particular reason. Therefore, DFAST, by default, removes biological features predicted at the edges of contigs or overlapping assembly gaps. This behavior can be modified via the configuration file.

By using the custom reference database (D), we could decrease the number of CDSs for proteins without any assigned function (hypothetical proteins or uncharacterized proteins). However, it was still larger than that by the former version (A). This is because CDSs flagged as possible pseudogenes (due to internal stop codons/frameshifts or partial hits against the reference sequences) are annotated with the protein name “hypothetical protein” in the new version. Even in that case, DFAST reports the hit coverages against the reference sequences, protein names inferred from the hits, and estimated positions of stop codons or frameshifts as reference information.

**Table S2. Comparison of annotation for *Lactobacillus parakefiri* JCM 8573^T^**

A) Annotated using the former version of DFAST (based on Prokka) with a custom protein database for lactic acid bacteria (GCA_002157585.1)

B) Annotated by PGAP (GCF_002157585.1)

C, D) Annotated by DFAST-core with the default reference database (C) and with a custom protein database for lactic acid bacteria (D)

E) Annotated by Prokka with its default reference database, F) Annotated by MiGAP with default settings

| Data source / Annotation tool | (A) INSDC | (B) RefSeq | (C) DFAST | (D) DFAST | (E) Prokka | (F) MiGAP |
| --- | --- | --- | --- | --- | --- | --- |
| Total CDS | 2441 | 2453 | 2442 | ← | 2444 | 2476 |
| *Pseudogene** | - | 273 (126/147) | 276 (134/142) | 258 (173/85) | [45**] | - |
| *with COG number* | - | - | 1449 | ← | - | 2211 |
| *unknown function* | 564 | 523 | 877 | 761 | 929 | 577 |
| tRNA | 63 | 63 | 63 | ← | 63 | 57 |
| rRNA | 4 | 26 | 4 | ← | 4 | 4 |
| CRISPR arrays | 2 | 1 | 2 | ← | 2 | - |
| Running time | - | - | 1m52s | 1m28s | 1m12s | 11h15m |

* Numbers in parentheses denote internal stop codon/frameshift and partial genes, respectively; ** Candidates for pseudogenes are mentioned in the log file, not in the result.

## *Candidatus* Brocadia sinica

We take *Candidatus* Brocadia sinica str. JPN1 (4.07Mb, GCA_000949635.1) from phylum *Planctomycetes* as an example of less well-characterized organisms (Oshiki *et al.*, 2015). So far, only 2 genomes have been registered at RefSeq for this genus. The RefSeq genome of this strain, which was automatically annotated by PGAP, contains many hypothetical proteins that account for as much as 57% of the predicted CDSs (GCF_000949635.1, Table S3-B). The default workflow of DFAST reported 2,109 hypothetical proteins (57%) (C), which was on the same level with PGAP but was much worse than that of the original annotation (A) or than those obtained from Prokka (H) and MiGAP (I), almost lacking annotation for its unique metabolic pathway called anammox. In addition, the number of CDSs labeled as ‘partial’ was obviously large (683 CDSs). Presumably, many of the sequences in the default database were from distant organisms, and only small portions were aligned to the query. As for the comparison with other pipelines, the current version of Prokka does not seem to consider the hit coverage. Therefore, users should pay attention to whether the predicted proteins are really intact or functional. MiGAP conducts homology search against more comprehensive databases such as UniProtKB/TrEMBL. Generally, MiGAP tend to annotate aggressively, which is useful for exploring gene functions in less well-studied species. However, some of the protein names are not appropriate and requires manual curation before being submitted to the INSDC.

There are several options to improve the annotation. One simple way is to decrease the threshold for the hit coverage at the risk of reliability. If it is set to 0, meaning that the hit coverage is no longer considered, the number of hypothetical proteins will become 1,503 (data not shown), which is much better than that by the default settings (C). The more plausible way is to provide references from closely-related species. DFAST has an option for orthologous assignment, which conducts all-against-all protein comparison between given reference genomes. We employed the draft genome of *Ca*. B. sapporoensis as a reference (GCF_001753675.2), yielding slightly a better result (D). Using a more comprehensive reference database is also effective. We used UniRef50 downloaded from the UniPprotKB (<http://www.uniprot.org/help/uniref)> as a reference and obtained a much better result (E). As the database size was large (23,133,394 sequences with 7,059,773,235 total residues) for GHOSTX, which has a large memory footprint, we first used BLASTP and took about 25h with 43GB of memory requirements on a 4 core Linux server. When UniRef50 was used in combination with orthologous assignment, we obtained an even better result and the running time was reduced to 15h (F). Specifically, genes related to anammox were more accurately annotated; for example, 8 out of 10 copies of hydroxylamine/hydrazine oxidoreductase were successfully identified. By default, DFAST skips downstream search processes for CDSs that have significant hits in upstream processes. Thus, conducting orthologous assignment prior to homology search against a large-sized database is also effective in reducing computation time. Finally, we tested GHOSTX for database search against UniRef50 (G), which required 75GB memory and finished 50 times faster than when BLASTP was used. Among the search results of BLASTP and GHOSTX, 97% were hits against the same sequence or against those with the same name, and many of the others were against those with the same function but with the different names. If the machine power permits, we recommend using GHOSTX.

As shown in this example, we recommend users to provide additional references to improve annotation for less well-characterized species. When the reference genomes from close relatives are available, using them as reference for orthologous assignment is a convenient way. For users of the web version, which currently does not accept user-provided optional references, we recommend using the annotation editor incorporated in the web service to curate annotated gene function before the submission to the INSDC.

**Table S3. Comparison of annotation for *Candidatus* Brocadia sinica str. JPN1**

A) Original annotation by submitters (GCA_000949635.1, 2015), B) Annotated by PGAP (GCF_000949635.1)

C–G) Annotated by DFAST-core with the default reference database (C), with ortholog assignment against *Ca*. B. sapporoensis strain 40 (GCF_001753675.2) (D), with UniRef50 (23,133,394 sequences) as a reference database using BLASTP (E), with both ortholog assignment against *Ca*. B. sapporoensi and UniRef50 (F), same as E but with using GHOSTX (G)

H) Annotated by Prokka with the default reference database, I) Annotated by MiGAP

| Data source / Annotation tool | (A) INSDC | (B) RefSeq | (C) DFAST | (D) DFAST | (E) DFAST | (F) DFAST | (G) DFAST | (H) Prokka | (I) MiGAP |
| --- | --- | --- | --- | --- | --- | --- | --- | --- | --- |
| Total CDS | 3912 | 3745 | 3704 | ← | ← | ← | ← | 3830 | 3718 |
| *Pseudogene** | - | 106 (82/24) | 766 (83/683) | 600 (124/476) | 172 (71/101) | 195 (120/75) | 189 (81/108) | [36**] | - |
| *Selenoprotein* | - | 1 | 1 | ← | ← | ← | ← | - | - |
| *with COG number* | - | - | 1842 | ← | ← | ← | ← | - | 3234 |
| *unknown function* | 1469 | 2128 | 2109 | 1890 | 1526 | 1344 | 1521 | 1882 | 1622 |
| tRNA | 47 | 47 | 48 | ← | ← | ← | ← | 48 | 47 |
| rRNA | 3 | 3 | 3 | ← | ← | ← | ← | 3 | 2 |
| CRISPR arrays | - | 4 | 4 | ← | ← | ← | ← | 4 | - |
| Running time | - | - | 4m04s | 4m11s | 25h38m ^#^ | 15h23m ^#^ | 29m27s ^##^ | 2m25s | 16h31m |

* Numbers in parentheses denote internal stop codon/frameshift and partial genes, respectively; ** Candidates for pseudogenes are mentioned in the log file, not in the result.

^#^ Run on a Linux system with 4 CPUs and memory usage of 43GB; ^##^ Run on a Linux system with 4 CPUs and memory usage of 75GB.

## *Methanosarcina barkeri*

*Methanosarcina barkeri* is a representative species of the methanogenic archaea and also known to be the first organism in which the translation of the amber codon into the 22^nd^ amino acid, pyrrolysine, was described (Srinivasan *et al.*, 2002). As a showcase for archaeal genome annotation, we annotated the complete genome of *M. barkeri* str. Fusaro (4.84 Mb, GCA_000195895.1), which is the first genome published for this species (Maeder *et al.*, 2006).

The default workflow of DFAST (Table S4-C) resulted in seemingly many hypothetical proteins and partial genes. Therefore, we tested DFAST with options for orthologous assignment and an additional database (D–F). Similar to the example of *Ca*. Brocadia sinica, when additional references were provided, DFAST generated better results in terms of the number of CDSs assigned with function. We used a recently published genome of *M*. *barkeri* str. CM1 (Lambie *et al.*, 2015) as a reference for orthologous assignment. As a result, 2,751 CDSs were assigned with orthologous relationship, showing that it is effective for transferring annotation as well as comparison with the reference. The running times of DFAST under the 4 different conditions were less than 10 minutes, which were considerably slower than Prokka. This was attributable for the small size of Prokka’s default database for Archaea (1,995 sequences). Accordingly, the result by Prokka contained more hypothetical proteins (G).

Under all the 4 different conditions tested, DFAST identified 6 pyrrolysine-containing proteins. They are annotated as members of methylamine methyltransferase known as key enzymes in methylotrophic methanogenesis (Rother and Krzycki, 2010). While only 2 proteins were reported as pyrrolysine-containing in the original annotation of strain Fusaro (A), 6–9 pyrrolysine-containing proteins are present in recently-published genomes of *M. barkeri*. Of note, in the RefSeq data annotated using PGAP (B), they are marked as pseudogenes due to internal stop codons. Presumably, translation exception to pyrrolysine is not taken into account for the current workflow of PGAP; we could not find protein entries that contain pyrrolysine in the RefSeq database.

**Table S4. Comparison of annotation for *Methanosarcina barkeri str.* Fusaro**

A) Original annotation by submitters (GCA_000195895.1, 2005), B) Annotated by PGAP (GCF_000195895.1)

C–F) Annotated by DFAST-core with the default reference database (C), with ortholog assignment against *M. barkeri* str. CM1 (GCA_001027005.1) (D), with RefSeq-nonredundant-proteins of Archaea as a reference database (E), with both ortholog assignment and RefSeq-nonredundant-proteins (F)

G) Annotated by Prokka with the default reference database, H) Annotated by MiGAP

| Data source / Annotation tool | (A) INSDC | (B) RefSeq | (C) DFAST | (D) DFAST | (E) DFAST | (F) DFAST | (G) Prokka | (H) MiGAP |
| --- | --- | --- | --- | --- | --- | --- | --- | --- |
| Total CDS | 3625 | 3989 | 4003 | ← | ← | ← | 4032 | 4024 |
| *Pseudogene** | - | 230 (154/76) | 552 (206/346) | 494 (247/247) | 315 (183/131) | 357 (249/108) | [51**] | - |
| *pyrrolysine containing* | 2 | - | 6 | ← | ← | ← | - | - |
| *with COG number* | - | - | 2510 | ← | ← | ← | - | 3093 |
| *unknown function* | 1711 | 1542 | 1892 | 1573 | 1577 | 1346 | 2354 | 1896 |
| tRNA | 63 | 62 | 60 | ← | ← | ← | 59 | 61 |
| rRNA | 9 | 9 | 9 | ← | ← | ← | 9 | 6 |
| CRISPR arrays | - | 3 | 5 | ← | ← | ← | 4 | - |
| Running time | - | - | 4m51s | 3m14s | 9m10s ^#^ | 9m22s ^#^ | 1m47s | 18h14m |

* Numbers in parentheses denote internal stop codon/frameshift and partial genes, respectively; ** Candidates for pseudogenes are mentioned in the log file, not in the result.

^#^ Run on a Linux system with 4 CPUs and memory usage of 15GB.

# Customization of the pipeline

DFAST allows 2 levels of customization. Firstly, the simpler way is by using command line options. In most cases, that will do for beginners as DFAST is designed to work well with its default settings, although it is sometimes not sufficient for distantly-related species as shown above. The examples shown in this paper, excepting BLASTP search against UniRef50, were executed with the default settings and command line options to specify additional references. Secondary, DFAST accepts a user-defined configuration file, which allows more flexible customization. Users can freely choose structural annotation tools to use, change the type and order of the reference database to search against, and specify thresholds and options to pass to each annotation process. For example, users can choose gene prediction tools, such as tRNAscan-SE for tRNA, Prodigal for CDS, and RNAmmer for rRNA, instead of the default tools, although users need to install the binaries as they are not included in the software distribution.

In addition, the modular structure of the DFAST increases the extensibility and reusability of the software. As the program components of DFAST are implemented as Python modules, they can be reused in other programs or in future development. A good example for this is a utility script, which is bundled in the software distribution, to adjust the sequence origin of the circular chromosome so that the *dnaA* gene should be placed at the first position. It is developed based on the DFAST modules and runs both as a stand-alone program and as an optional step in the default DFAST pipeline.

The pipeline of Prokka can be customized through command line options. In addition to the options for additional reference databases, Prokka has options to enable signal peptide and ncRNA prediction. Currently, DFAST does not have functions to predict them, as we have still difficulty in curating those results into appropriate forms for INSDC submission. MiGAP has an option for expert users to choose reference databases to search against. MiGAP runs on the Super Computer System of National Institute of Genetics (Mishima, Japan), being suited for a large-scale database search such as the NCBI non-redundant proteins (nr) and UniProtKB/TrEMBL, which is difficult on a small-scale desktop or laptop computer.

# References

Lambie,S.C. *et al.* (2015) The complete genome sequence of the rumen methanogen Methanosarcina barkeri CM1. *Stand. Genomic Sci.*, **10**, 57.

Maeder,D.L. *et al.* (2006) The Methanosarcina barkeri genome: comparative analysis with Methanosarcina acetivorans and Methanosarcina mazei reveals extensive rearrangement within methanosarcinal genomes. *J. Bacteriol.*, **188**, 7922–7931.

Oshiki,M. *et al.* (2015) Draft Genome Sequence of an Anaerobic Ammonium-Oxidizing Bacterium, "Candidatus Brocadia sinica". *Genome Announc.*, **3**, e00267-15.

Rother,M. and Krzycki,J.A. (2010) Selenocysteine, pyrrolysine, and the unique energy metabolism of methanogenic archaea. *Archaea*, **2010**, 453642.

Srinivasan,G. *et al.* (2002) Pyrrolysine encoded by UAG in Archaea: charging of a UAG-decoding specialized tRNA. *Science*, **296**, 1459–1462.

Tanizawa,Y. *et al.* (2016) DFAST and DAGA: Web-based integrated genome annotation tools and resources. *Biosci Microbiota Food Health*, **35**, 173–184.

Tanizawa,Y. *et al.* (2017) Genomic characterization reconfirms the taxonomic status of Lactobacillus parakefiri. *Biosci Microbiota Food Health*, **36**, 129–134.
